# Mechanism study on the attenuation of cerebral ischemia–reperfusion injury by LBP extract through regulation of SIRT1/PGC-1α axis

**DOI:** 10.1515/tnsci-2025-0377

**Published:** 2025-07-11

**Authors:** Qingfeng Niu, Jiahui Peng, Yujia Zhou, Xiaowen Li, Ouya Liu, Cheng Xin, Ping Liu, Changchun Hei, Xiao Yang

**Affiliations:** Department of Neurology, General Hospital of Ningxia Medical University, Yinchuan, China; School of Basic Medicine, Ningxia Medical University, Yinchuan, China; Department of Endocrinology, General Hospital of Ningxia Medical University, Yinchuan, China; Peking University First Hospital-Ningxia Women Children Hospital, Yinchuan, China

**Keywords:** hypoxia-reperfusion, LBP, oxidative stress, SIRT1, PGC-1α, ROS

## Abstract

**Objective:**

This study aims to determine if *Lycium barbarum* polysaccharides (LBP) extract attenuate oxidative stress by regulating the SIRT1/PGC-1α axis, potentially ameliorating oxygen–glucose deprivation/reperfusion (OGD/R)-induced neuronal damage.

**Methods:**

A cellular hypoxia/reoxygenation model (OGD/R) using HT22 cells was established to simulate cerebral ischemia–reperfusion injury. Cells were allocated into four groups: normal (Control), hypoxia (OGD/R), LBP extract-treated (OGD/R + LBP at 25, 50, 100 μg/mL), and SIRT1-inhibited (OGD/R + S100). Western blot and qPCR were performed to detect the expression of pathway-related factors, oxidative stress, mitochondrial function, and apoptosis-related factors.

**Results:**

Compared to the Control group, the OGD/R group exhibited significantly reduced cell survival, increased LDH release, apoptosis rate, and reactive oxygen species (ROS) levels. After intervention with LBP extract, cell survival increased, LDH release, ROS levels, and apoptosis rates reduced. The above injuries were associated with the inhibition of the SIRT1/PGC-1α pathway. LBP extract can attenuate the hypoxia-reperfusion-induced inhibition of the SIRT1/PGC-1α pathway and reverse the resulting high levels of oxidative stress and apoptosis, ultimately ameliorating cellular injury.

**Conclusion:**

LBP extract’s protective effects against ischemia–reperfusion injury in HT22 cells appear linked to the modulation of the SIRT1/PGC-1α pathway and a reduction in oxidative stress.

## 1 Introduction

Stroke is a leading disease, significantly endangering human life and health due to high incidence, disability, recurrence, and mortality rates. Ischemic strokes constitute approximately 75% of all stroke cases [1,2]. The primary goal of acute ischemic stroke treatment is timely restoration of cerebral perfusion, commonly achieved via drug thrombolysis and arterial thrombolysis [3,4]. Despite the efficacy of these treatments in restoring blood flow, they require a strict time window, and the risk of secondary cerebral ischemia–reperfusion injury (CIRI) remains even after recirculation.

The pathogenesis of CIRI is multifactorial, involving oxidative stress, leukocyte infiltration, platelet adhesion and aggregation, complement activation, apoptosis, and blood–brain barrier impairment. These factors ultimately result in adverse events such as cerebral edema, herniation, and hemorrhagic transformation, which significantly worsen patient prognosis. Oxidative stress represents a primary pathological mechanism within this process, as reperfusion causes a surge in reactive oxygen species (ROS) production, far exceeding cellular clearance capacities, thus triggering downstream cascades that exacerbate brain tissue injury [5].

Silent information regulator 1 (SIRT1), a nicotinamide adenine dinucleotide (NAD+)-dependent deacetylase, regulates peroxisome proliferator-activated receptor coactivator 1 alpha (PGC-1α), enhancing its transcriptional activity upon deacetylation. Research indicates that SIRT1 is primarily expressed in cerebrovascular endothelial and smooth muscle cells, as well as in neurons across regions like the cortex, hippocampus, and hypothalamus [6], providing a foundation for SIRT1’s regulatory roles in cerebral vasculature, energy metabolism, and pathophysiological changes following ischemia [7]. The SIRT1/PGC-1α signaling pathway modulates processes including cellular senescence, oxidative stress, glycolipid metabolism, angiogenesis, and inflammation, closely linking it to both apoptosis and oxidative stress. Activation of PGC-1α further influences nuclear respiratory factors, estrogen-related receptors, myocyte enhancer factor-2c, and proteins such as mitochondrial transcription factor A (TFAM), promoting mitochondrial biogenesis, glucose utilization, and fatty acid oxidation, which exert neuroprotective effects [8]. Additionally, upregulation of SIRT1 has been shown to enhance neuronal tolerance to ROS toxicity [9–12].

*Lycium barbarum* (wolfberry) contains various active compounds, with *Lycium barbarum* polysaccharide (LBP) as the primary bioactive component with high medicinal value. Studies demonstrate that LBP exhibits diverse biological activities, including hepatoprotection, anti-tumor effects, immune modulation, glycemic and lipid regulation, antioxidant properties, and anti-aging effects [13,14]. Moreover, it is safe, non-toxic, cost-effective, and possesses potential clinical applications. Notably, LBP’s organ-protective effects largely stem from its antioxidant properties, which include scavenging free radicals, enhancing enzymatic activities, and activating antioxidative stress pathways.

Prior studies indicate that LBP extract attenuates oxygen–glucose deprivation/reperfusion (OGD/R)-induced cellular injury. Given the importance of the SIRT1/PGC-1α signaling pathway in apoptosis and oxidative stress, and the antioxidative potential of LBP extract, we hypothesize that LBP extract could mitigate oxidative stress via the SIRT1/PGC-1α pathway, thereby attenuating CIRI. This study assesses apoptosis and oxidative stress by constructing a cellular hypoxia/reoxygenation model to determine whether LBP extract reduces hypoxia-reperfusion injury in HT22 cells by modulating mitochondrial oxidative stress and apoptosis. Additionally, we employed the SIRT1-specific inhibitor EX-527 (Selisistat) to explore whether LBP extract’s antioxidant effects are mediated through the SIRT1/PGC-1α pathway, thereby conferring cerebroprotective effects.

## 2 Materials and methods

### 2.1 Experimental cells

The HT22 mouse hippocampal neuronal cell line was obtained from Shanghai Enzyme-linked Biotechnology Co., Ltd.

### 2.2 Experimental reagents

LBP with a purity >50% was sourced from Beijing Solarbio Science & Technology Co., Ltd. LBP was directly dissolved in the culture medium to create a 1 mg/mL stock solution. Dulbecco's modified Eagle Medium (DMEM) high-glucose medium and fetal bovine serum (FBS) were sourced from Biological Industries Ltd; trypsin, penicillin, and streptomycin were obtained from Beijing Solarbio Science & Technology Co., Ltd; the Enhanced Cell Counting Kit-8 (C0042), ROS Assay Kit with CM-H2DCFDA (S0033M), and LDH Assay Kit with WST-8 (C0018S) were obtained from Beyotime; the Annexin V-APC/7-AAD Apoptosis Kit (KGA1106-10) from KeyGen BioTECH; TB Green Premix Ex Taq II (RR820A) from Takara Bio; EX-527 (Selisistat, SC2081-10mM) from Beyotime; and antibodies for SIRT1 (2028S), PGC-1α (2178S), NRF1 (46743S), TFAM (8076S), Bax (2772S), Caspase-3 (9662S), Bcl-2 (3498S), β-actin (4967S), and H3 (9715S) were sourced from Cell Signaling Technology.

### 2.3 Cell culture

HT22 cells were maintained in DMEM high-glucose complete medium supplemented with 10% FBS and 1% penicillin–streptomycin. Cells were seeded in culture flasks and incubated at 37°C with 5% CO_2_. When cell confluency reached approximately 80%, the cells were washed twice with PBS, digested with trypsin, centrifuged, and resuspended in fresh medium for further culture. Medium was refreshed every other day, and passaging occurred every 3 days. Cells in the logarithmic growth phase were used for experiments.

### 2.4 Cell grouping and OGD/R model

HT22 cells were seeded in six-well plates and introduced to DMEM glucose-free medium at approximately 60% confluency. They were cultured in a hypoxic incubator (94% N2, 5% CO_2_, and 1% O_2_) for 2 h to induce hypoxia. After hypoxia, the medium was replaced with DMEM containing 10% FBS, and cells were incubated for 24 h to establish the *in vitro* OGD/R model. Cells received LBP extract intervention or LBP extract with the SIRT1-specific inhibitor EX-527 (Selisistat, 1 μM). Groups included Control, OGD/R, OGD/R + LBP (25, 50, and 100 μg/mL), and OGD/R + S100. The LBP extract concentration gradient was determined based on existing literature [13,15].

Cells were plated in 96-well plates at a density of 4,000 cells per well, with 100 μL of suspension per well, and five replicates per condition. After reoxygenation, cells were washed with PBS and treated with 10% cell counting kit-8 (CCK8) solution (90% DMEM, 10% CCK8), adding 100 μL to each well. A set of blank wells (without cells) was included to correct for media effects. After 2 h, absorbance at 450 nm was measured. Cell survival rate was calculated as (OD experimental group – OD blank)/(OD control – OD blank) × 100%.

### 2.5 Lactate dehydrogenase (LDH) release assay

Cells were plated in 96-well plates at 4,000 cells per well, with wells for sample maximum enzyme activity, control, and modeled conditions (OGD/R and LBP extract concentrations). LDH releasing agent (10 μL) was added to maximum enzyme activity wells and incubated for 1 h. The plate was centrifuged at 400*g* for 5 min, and 120 μL of supernatant from each well was transferred to a new plate for LDH determination. LDH working solution was prepared and 60 μL was added to each well, incubated for 30 min at room temperature, and absorbance was measured at 490 nm. Cytotoxicity or mortality (%) = (absorbance of treated samples – absorbance of sample control wells)/(absorbance of maximum enzyme activity – absorbance of sample control wells) × 100%.

### 2.6 Flow cytometry apoptosis detection

Cells were seeded in six-well plates at 100,000 cells per well, with treatments applied at 60% confluency. After reoxygenation, cells were trypsinized, washed with PBS, and resuspended in 500 μL binding buffer. Five microliters of APC and 7-AAD were added, gently mixed, incubated for 10 min at room temperature in the dark, and analyzed via flow cytometry (BD Accuri C6) within 30 min.

### 2.7 Flow cytometry ROS assay

Cells were seeded in six-well plates at 100,000 cells per well and treated as per group. 2′,7′-dichlorodihydrofluorescein diacetate (DCFH-DA) working solution (10 μmol/L) was prepared in serum-free DMEM. After incubation, cells were stained, washed, resuspended in PBS, and analyzed by flow cytometry.

### 2.8 Real-time quantitative PCR (RT-PCR)

RNA was extracted post-reoxygenation and reverse-transcribed using PrimeScript RT Kit. PCR was performed with a 25 μL reaction volume at conditions of 95°C for 5 s and 60°C for 30 s (40 cycles). Gene expression differences were analyzed using the 2^−∆∆Ct^ method. The primer sequences are detailed in Table 1. Before performing RT-PCR, the concentration and purity of RNA are measured using spectrophotometry. An A260/A280 ratio between 1.8 and 2.0 is considered indicative of a pure RNA sample. Designing primers using SnapGene software with the following parameters: *T*_m_: 55–65°C, length: 18–27 bp, and GC content: 40–60%.

**Table 1:** Primer sequences

Name	Forward primer (5′−3′)	Reverse primer (5′−3′)
SIRT1	TGAGACCAGTAGCACTAATTCCAA	CTCCAAGGAGCTCTACATCAAAAT
PGC-1α	GAAAAAGAAGTCCCATACACAACC	GTTCGCTCAATAGTCTTGTTCTCA
TFAM	TCGATTTTCCACAGAACAGCTAC	CCACTCAGCTTTAAAATCAGCTTC
NRF1	TCACAGACCGTAGTACAGACCTTC	AGAGTAATTCACTTGGGCAACAGT
GAPDH	GGTTGTCTCCTGCGACTTCA	TGGTCCAGGGTTTCTTACTCC

### 2.9 Western blot analysis

Cells were seeded in six-well plates and treated at 60% confluency. Protein was extracted and quantified via BCA. Samples (30 μg) underwent SDS-PAGE electrophoresis (5% stacking gel at 80 V, 10% separation gel at 120 V), wet transferred to membranes, blocked for 1 h, and incubated with primary and secondary antibodies. Band density was analyzed using ImageJ.

### 2.10 Statistical methods

Data were analyzed with SPSS23 and GraphPad Prism 6 software. Normal distribution data were compared using one-way ANOVA or Chi-square for multiple groups and *t*-tests for two-group comparisons, with significance set at *P* < 0.05.

**Figure 1:**
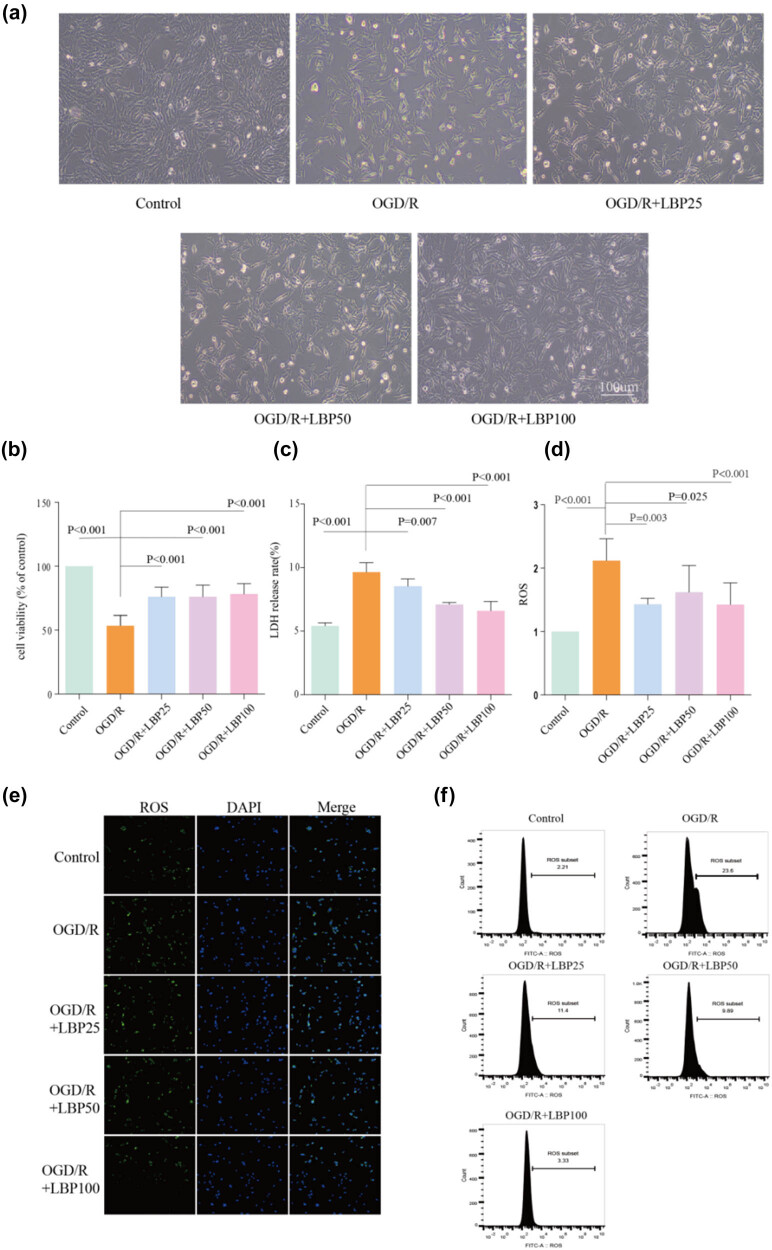
Effects of LBP extract on HT22 neuronal cells under OGD/R conditions. (a) Morphological changes of HT22 cells observed under an inverted microscope (scale bar = 100 μm). (b) Assessment of HT22 cell viability using the CCK8 assay. (c) Measurement of LDH release rate across groups. (e) Immunofluorescent detection of intracellular ROS levels. (d) and (f) Flow cytometry analysis of intracellular ROS levels.

## 3 Results

### 3.1 Effect of LBP extract on morphology and survival of OGD/R-damaged neuronal cells

(1) HT22 cell morphology was observed under an inverted microscope, revealing significant changes in the OGD/R group compared to the Control group (Figure 1a). Cells in the OGD/R group exhibited reduced density, unclear outlines, oval or spindle shapes, an increased number of floating cells, shortened neuronal synapses, and a reduced refractive index. In the LBP extract-treated groups (25, 50, and 100 μg/mL), cell density increased, the number of floating cells decreased, cell outlines became clearer, and most cells appeared round, with fewer retaining an oval or spindle shape. Neuronal projections were more elongated and intertwined to form a network, with enhanced refractive properties.
(2) Cell viability was assessed via the CCK8 assay, showing a significant decrease in the OGD/R group compared to the Control group (*P* < 0.001, Figure 1b). In contrast, LBP extract-treated groups (25, 50, and 100 μg/mL) displayed significantly increased cell viability compared to the OGD/R group (*P* < 0.001), with the highest viability observed in the OGD/R + LBP (100 μg/mL) group.

### 3.2 Effect of LBP extract on LDH release rate in OGD/R-injured neurons

Neuronal damage was further assessed by measuring LDH release, showing (Figure 1c) that the LDH release rate significantly increased in the OGD/R group compared to the Control group (*P* < 0.001). LBP extract treatment at 25, 50, and 100 μg/mL led to significant reductions in LDH release compared to the OGD/R group (*P* = 0.007, *P* < 0.001, *P* < 0.001, respectively), with the lowest LDH release observed in the OGD/R + LBP (100 μg/mL) group.

### 3.3 Effect of LBP extract on ROS levels in OGD/R-injured neurons

Intracellular ROS levels were determined by DCFH-DA fluorescent probe labeling, with ROS distribution observed under a confocal microscope (Figure 1e) and quantified via flow cytometry (Figure 1d and f). The OGD/R group exhibited significantly higher ROS levels than the Control group (*P* < 0.001). In contrast, LBP extract-treated groups (25, 50, and 100 μg/mL) showed significantly reduced ROS levels compared to the OGD/R group (*P* = 0.003, *P* = 0.025, *P* < 0.001, respectively).

### 3.4 Effect of LBP extract on apoptosis levels in OGD/R-injured neurons

**Figure 2:**
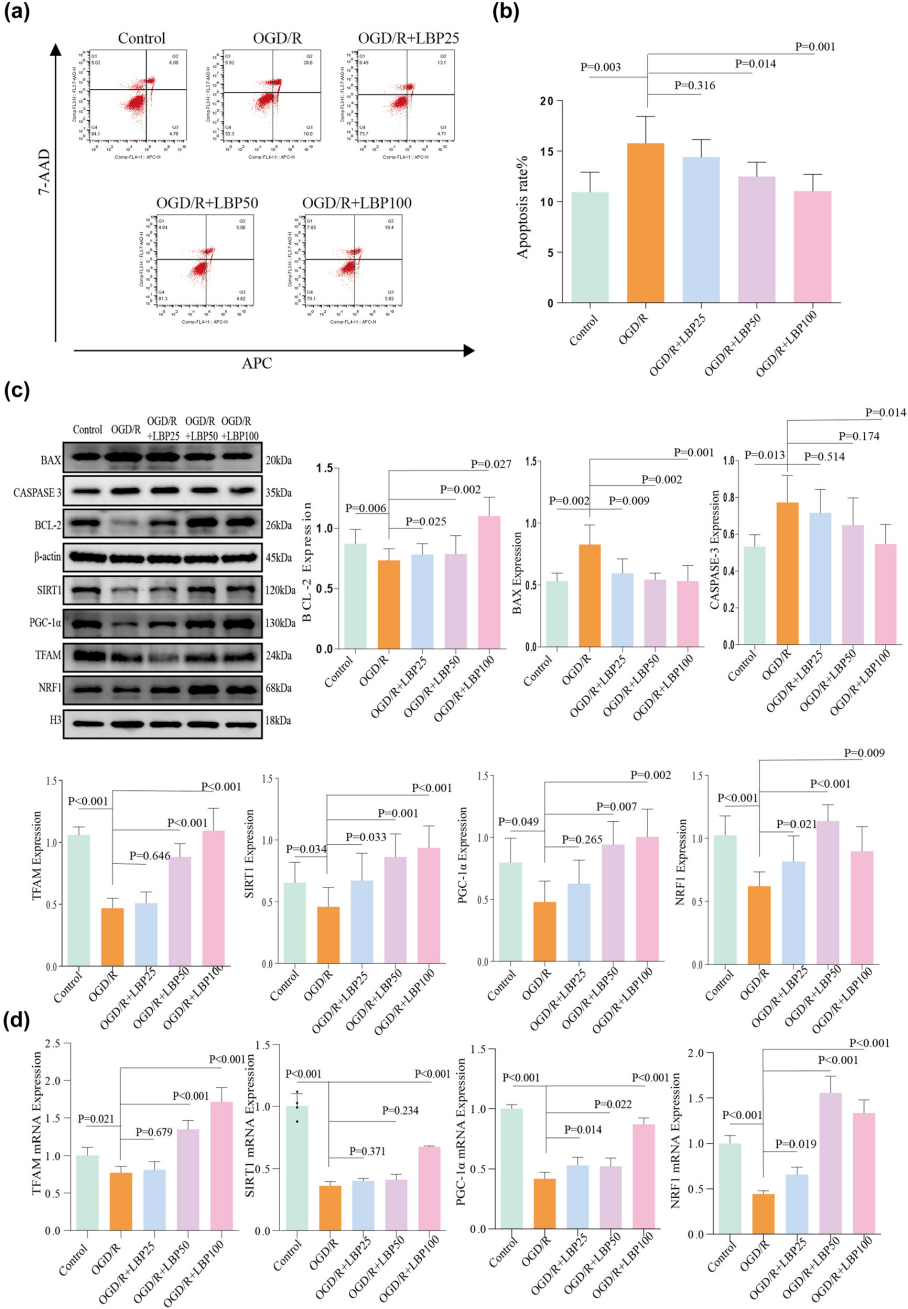
Effects of LBP extract on apoptosis, protein expression, and gene expression in HT22 neuronal cells under OGD/R conditions. (a) Flow cytometry analysis of apoptosis in HT22 cells across different treatment groups. (b) Quantitative analysis of apoptosis rates for each group, represented as a histogram. (c) Western blot analysis of protein expression levels related to the SIRT1/PGC-1α pathway, oxidative stress, and apoptosis in HT22 cells. (d) qPCR analysis of gene expression levels for key factors involved in the SIRT1/PGC-1α pathway and related downstream targets.

Flow cytometry was employed to assess apoptosis levels in HT22 cells post-hypoxia-reperfusion (Figure 2a and b). Apoptosis rates significantly increased in the OGD/R group compared to the Control group (*P* = 0.003). In the LBP extract-treated groups (50 and 100 μg/mL), apoptosis rates were significantly reduced compared to the OGD/R group (*P* = 0.014, *P* = 0.001, respectively), while the 25 μg/mL LBP extract group did not show a statistically significant difference (*P* = 0.316). Results indicated that the optimal LBP extract concentration for reducing cell damage in OGD/R conditions was 100 μg/mL.

### 3.5 Effects of LBP extract on SIRT1/PGC-1α signaling pathway, oxidative stress, mitochondrial function, and apoptosis-related molecules in OGD/R-injured neurons

The expression levels of proteins involved in the SIRT1/PGC-1α signaling pathway, oxidative stress markers, mitochondrial function, and apoptosis were assessed via western blot (Figure 2c):

(1) SIRT1/PGC-1α pathway: Protein levels of SIRT1 and PGC-1α were significantly reduced in the OGD/R group compared to the Control group (*P* = 0.034, *P* = 0.049, respectively). In the LBP extract-treated groups (25, 50, and 100 μg/mL), SIRT1 levels significantly increased compared to the OGD/R group (*P* = 0.033, *P* = 0.001, *P* < 0.001, respectively). PGC-1α expression similarly increased at 50 and 100 μg/mL (*P* = 0.007, *P* = 0.002), though no significant elevation was observed at 25 μg/mL (*P* = 0.265).
(2) Oxidative stress marker: The expression of NRF1, an oxidative stress marker, was significantly reduced in the OGD/R group compared to the Control group (*P* < 0.001). However, NRF1 levels significantly increased in LBP extract-treated groups at 25, 50, and 100 μg/mL compared to the OGD/R group (*P* = 0.021, *P* < 0.001, *P* = 0.009, respectively).
(3) Mitochondrial function: Expression of TFAM, a mitochondrial function marker, was significantly lower in the OGD/R group compared to the Control group (*P* < 0.001). TFAM expression significantly increased in the LBP extract-treated groups at 50 and 100 μg/mL compared to the OGD/R group (*P* < 0.001), but no significant change was observed at 25 μg/mL (*P* = 0.646).
(4) Apoptosis-related proteins: The anti-apoptotic protein Bcl-2 was significantly reduced in the OGD/R group compared to the Control group (*P* = 0.006). Bcl-2 levels significantly increased in LBP extract-treated groups at 25, 50, and 100 μg/mL (*P* = 0.025, *P* = 0.002, *P* = 0.027, respectively) compared to the OGD/R group. The pro-apoptotic proteins Bax and Caspase-3 were significantly elevated in the OGD/R group relative to the Control group (*P* = 0.002, *P* = 0.013, respectively). Bax expression was significantly reduced in LBP extract-treated groups (25, 50, and 100 μg/mL) compared to the OGD/R group (*P* = 0.009, *P* = 0.002, and *P* = 0.001). Caspase-3 expression was significantly reduced only in the 100 μg/mL LBP extract-treated group (*P* = 0.014), with no significant reduction observed at 25 or 50 μg/mL (*P* = 0.514, *P* = 0.174).

These findings suggest that LBP extract may exert protective effects against OGD/R-induced damage in HT22 cells through the SIRT1/PGC-1α signaling pathway, potentially mediated by antioxidative stress, anti-apoptotic effects, and preservation of mitochondrial function.

### 3.6 Effects of LBP extract on SIRT1/PGC-1α signaling pathway and downstream TFAM and NRF1 gene expression in OGD/R-injured neurons

To investigate the molecular mechanisms of LBP extract in alleviating OGD/R-induced injury, PCR was conducted to quantify the gene expression of SIRT1, PGC-1α, TFAM, and NRF1 (Figure 2d):

(1) SIRT1 and PGC-1α gene expression: Gene expression levels of SIRT1 and PGC-1α were significantly reduced in the OGD/R group compared to the Control group (*P* < 0.001). In the LBP extract-treated groups, PGC-1α expression levels significantly increased at all concentrations (25, 50, and 100 μg/mL; *P* = 0.014, *P* = 0.022, *P* < 0.001, respectively). However, SIRT1 expression levels did not significantly change in the 25 and 50 μg/mL groups (*P* = 0.371, *P* = 0.234), but significantly increased in the 100 μg/mL group (*P* < 0.001).
(2) TFAM and NRF1 gene expression: TFAM and NRF1 gene expression were significantly lower in the OGD/R group compared to the Control group (*P* = 0.021, *P* < 0.001). In LBP extract-treated groups, TFAM expression significantly increased in the 50 and 100 μg/mL groups (*P* < 0.001), but showed no significant change at 25 μg/mL (*P* = 0.679). NRF1 gene expression significantly increased across all LBP extract concentrations (25, 50, and 100 μg/mL; *P* = 0.019, *P* < 0.001, *P* < 0.001, respectively).

These results further support the hypothesis that LBP extract mitigates OGD/R-induced neuronal injury through upregulation of the SIRT1/PGC-1α signaling pathway and its downstream targets, TFAM and NRF1, contributing to antioxidative and neuroprotective effects.

**Figure 3:**
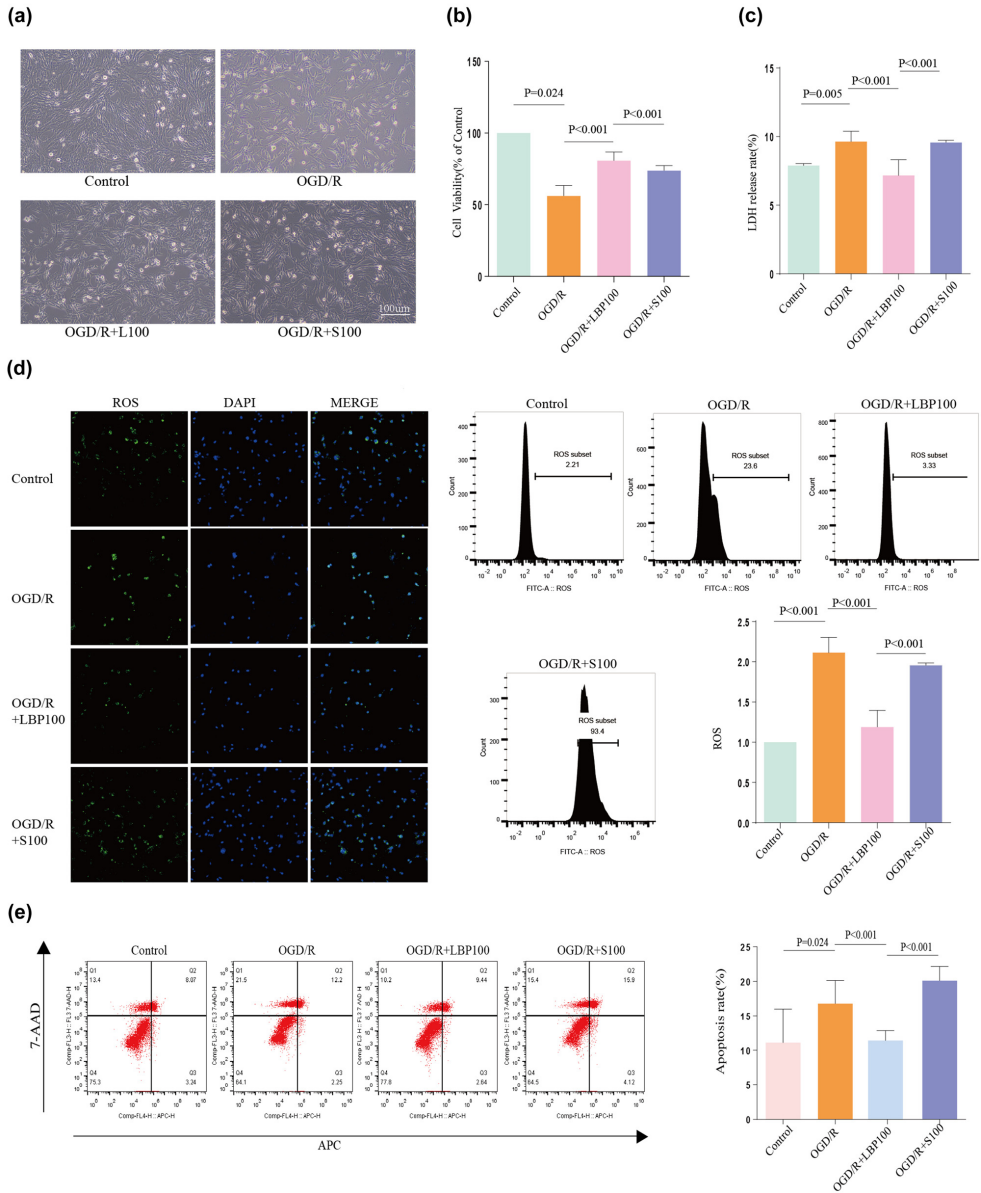
Effects of SIRT1 inhibition on HT22 cell morphology, viability, LDH release, ROS levels, and apoptosis following LBP extract treatment under OGD/R conditions. (a) Light microscopy images of HT22 cell morphology across treatment groups. (b) Cell viability assessment using the CCK8 assay across different groups. (c) Measurement of LDH release rates in each treatment group to evaluate cellular damage. (d) Confocal microscopy and flow cytometry analysis of ROS levels, with accompanying statistical histogram. (e) Flow cytometry analysis of apoptosis rates across groups, along with a statistical histogram.

### 3.7 Effect of SIRT1 inhibition on cell morphology and survival of neuronal OGD/R after LBP extract treatment

(1) Cell morphology: Cell morphology observed under an inverted microscope (Figure 3a) showed that, compared to the Control group, cells in the OGD/R group exhibited unclear outlines, an increased number of floating cells, shortened neuronal synapses, and a reduced refractive index. In the OGD/R + LBP (100 μg/mL) group, cell density increased, floating cells decreased, cell outlines were clearer, and most cells appeared rounded with elongated protrusions, some of which interwove into a network, enhancing refractive properties. However, in the OGD/R + S100 group (with SIRT1 inhibition), cells displayed more elliptical or spindle shapes, and synapses were shortened, suggesting a partial reversal of LBP extract’s morphological protection.
(2) Cell survival rate: Cell survival rate, assessed via CCK8 assay (Figure 3b), showed that survival was significantly reduced in the OGD/R group compared to the Control group (*P* = 0.024). The OGD/R + LBP (100 μg/mL) group displayed a significant increase in cell survival compared to the OGD/R group (*P* < 0.001). In contrast, the OGD/R + S100 group exhibited a significant reduction in survival compared to the OGD/R + LBP (100 μg/mL) group (*P* < 0.001), indicating that the SIRT1 inhibitor, EX-527 (Selisistat), reversed the protective effects of LBP extract in OGD/R cells.

### 3.8 Effect of SIRT1 inhibition on LDH release rate in LBP extract-treated OGD/R cells

Neuronal damage was further assessed by measuring the LDH release rate (Figure 3c). Compared to the Control group, LDH release was significantly higher in the OGD/R group (*P* = 0.005). LBP extract treatment (100 μg/mL) significantly reduced LDH release compared to the OGD/R group (*P* < 0.001). However, LDH release significantly increased in the OGD/R + S100 group compared to the OGD/R + LBP (100 μg/mL) group (*P* < 0.001), suggesting that EX-527 could reverse the effect of LBP extract in reducing cellular damage in OGD/R cells.

### 3.9 Effect of SIRT1 inhibition on ROS levels in LBP extract-treated OGD/R cells

Intracellular ROS levels were assessed using DCFH-DA fluorescent probe labeling, with ROS distribution visualized under a confocal microscope, and quantified via flow cytometry (Figure 3d). ROS levels were significantly elevated in the OGD/R group compared to the Control group (*P* < 0.001). The OGD/R + LBP (100 μg/mL) group displayed significantly lower ROS levels compared to the OGD/R group (*P* < 0.001). In contrast, the OGD/R + S100 group had significantly elevated ROS levels compared to the OGD/R + LBP (100 μg/mL) group (*P* < 0.001), indicating that SIRT1 inhibition mitigated the ROS-reducing effects of LBP extract on OGD/R-induced oxidative stress injury.

### 3.10 Effect of SIRT1 inhibition on apoptosis in LBP extract-treated OGD/R cells

The apoptosis rate, determined via flow cytometry (Figure 3e), showed that apoptosis was significantly elevated in the OGD/R group compared to the Control group (*P* = 0.024). Treatment with LBP extract (100 μg/mL) significantly reduced apoptosis in the OGD/R + LBP group compared to the OGD/R group (*P* < 0.001). However, in the OGD/R + S100 group, apoptosis was significantly higher than in the OGD/R + LBP (100 μg/mL) group (*P* < 0.001), indicating that EX-527 reversed the LBP extract-mediated reduction in apoptosis caused by OGD/R.

### 3.11 Effects of SIRT1 inhibition on SIRT1/PGC-1α signaling pathway, oxidative stress, and mitochondrial function in LBP extract-treated OGD/R neurons

**Figure 4:**
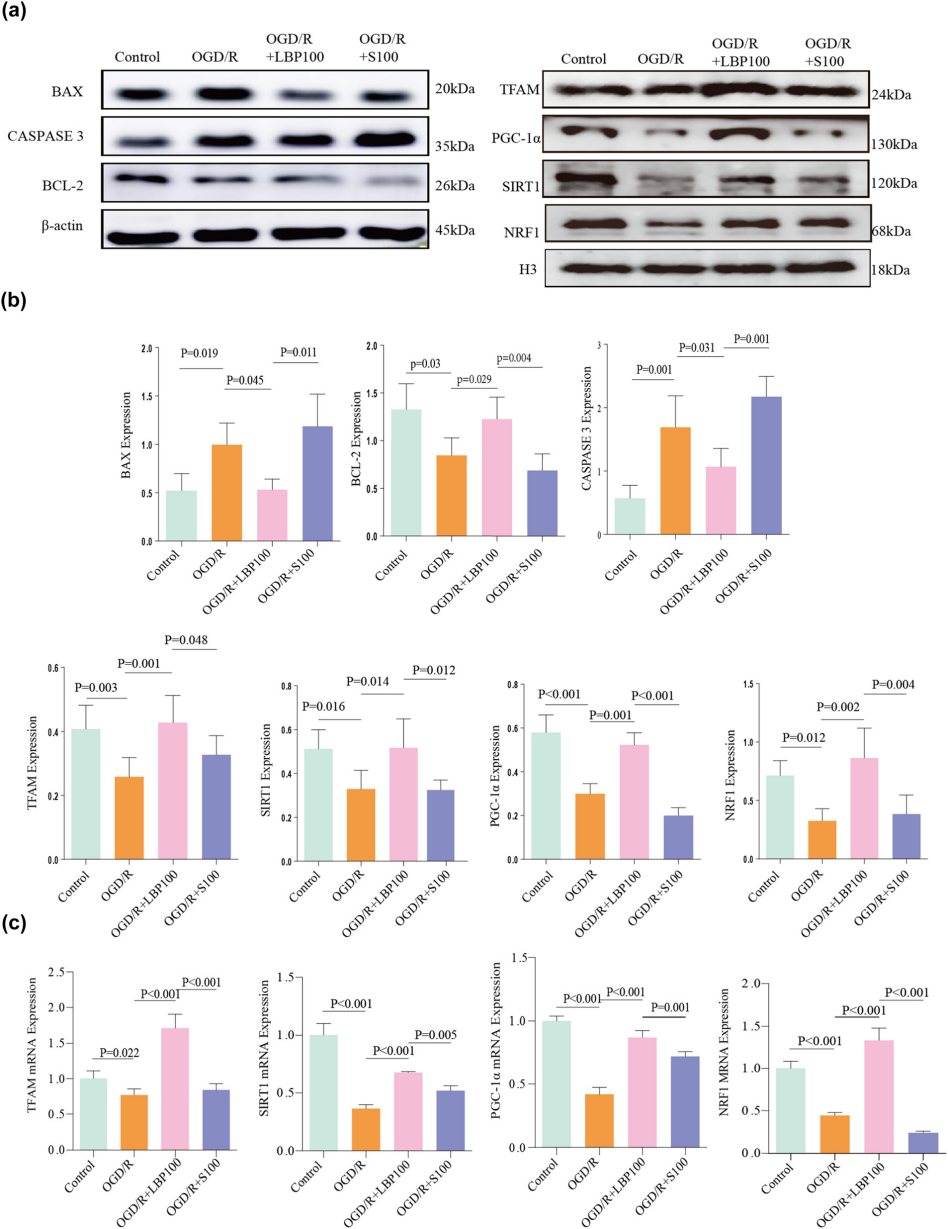
Effects of SIRT1 inhibition on protein and gene expression in LBP extract-treated OGD/R-injured neurons. (a) Western blot analysis showing protein bands for cell-associated proteins in each treatment group following SIRT1 inhibition. (b) Quantitative analysis of protein expression levels across groups, represented as a statistical graph. (c) Statistical histogram of related gene expression levels as measured by qPCR across different treatment groups.

The expression levels of proteins in the SIRT1/PGC-1α signaling pathway, markers of oxidative stress, and apoptosis-related proteins were assessed via western blot (Figure 4a and b):

(1) SIRT1 and PGC-1α pathway: SIRT1 and PGC-1α protein levels were significantly reduced in the OGD/R group compared to the Control group (*P* = 0.016, *P* < 0.001). Treatment with LBP extract (100 μg/mL) in the OGD/R + LBP group significantly elevated SIRT1 and PGC-1α expression compared to the OGD/R group (*P* = 0.014, *P* = 0.001). However, in the OGD/R + S100 group, which included SIRT1 inhibition, SIRT1 and PGC-1α levels were significantly reduced compared to the OGD/R + LBP (100 μg/mL) group (*P* = 0.012, *P* < 0.001).
(2) Oxidative stress marker (NRF1): The oxidative stress marker NRF1 was significantly reduced in the OGD/R group compared to the Control group (*P* = 0.012). In the OGD/R + LBP (100 μg/mL) group, NRF1 levels were significantly higher than in the OGD/R group (*P* = 0.002). However, NRF1 expression was significantly lower in the OGD/R + S100 group compared to the OGD/R + LBP (100 μg/mL) group (*P* = 0.004), indicating that SIRT1 inhibition reversed the LBP extract-induced increase in NRF1.
(3) Mitochondrial function (TFAM): TFAM, a mitochondrial function-related protein, was significantly lower in the OGD/R group compared to the Control group (*P* = 0.003). In the OGD/R + LBP (100 μg/mL) group, TFAM levels increased significantly compared to the OGD/R group (*P* = 0.001). However, TFAM expression significantly decreased in the OGD/R + S100 group compared to the OGD/R + LBP (100 μg/mL) group (*P* = 0.048).
(4) Apoptosis-related proteins: The anti-apoptotic protein Bcl-2 was significantly reduced in the OGD/R group compared to the Control group (*P* = 0.03). Bcl-2 expression was elevated in the OGD/R + LBP (100 μg/mL) group compared to the OGD/R group (*P* = 0.029), but decreased significantly in the OGD/R + S100 group compared to the OGD/R + LBP (100 μg/mL) group (*P* = 0.004). Pro-apoptotic proteins Bax and Caspase-3 were significantly elevated in the OGD/R group compared to the Control group (*P* = 0.019, *P* = 0.001, respectively). Bax and Caspase-3 levels were lower in the OGD/R + LBP (100 μg/mL) group compared to the OGD/R group (*P* = 0.045, *P* = 0.031), yet increased in the OGD/R + S100 group compared to the OGD/R + LBP (100 μg/mL) group (*P* = 0.011, *P* = 0.001).

These findings suggest that LBP extract protects HT22 cells against OGD/R injury through antioxidative effects, mitochondrial preservation, and apoptosis reduction, likely mediated by the SIRT1/PGC-1α signaling pathway. Notably, SIRT1 inhibition markedly weakened the protective effects of LBP extract, further supporting its neuroprotective role through the SIRT1/PGC-1α pathway.

### 3.12 Effect of SIRT1 inhibition on SIRT1/PGC-1α signaling pathway and downstream TFAM and NRF1 gene expression in LBP extract-treated OGD/R-injured neurons

PCR was used to measure the gene expression of SIRT1, PGC-1α, TFAM, and NRF1 to further evaluate the effects of SIRT1 inhibition on LBP extract-treated cells (Figure 4c):

(1) SIRT1 and PGC-1α gene expression: SIRT1 and PGC-1α gene expression significantly decreased in the OGD/R group compared to the Control group (*P* < 0.001). In the OGD/R + LBP (100 μg/mL) group, SIRT1 and PGC-1α expression levels significantly increased compared to the OGD/R group (*P* < 0.001). However, SIRT1 and PGC-1α gene expression was significantly lower in the OGD/R + S100 group than in the OGD/R + LBP (100 μg/mL) group (*P* = 0.005, *P* = 0.001).
(2) TFAM and NRF1 gene expression: TFAM and NRF1 expression levels were significantly lower in the OGD/R group compared to the Control group (*P* = 0.022, *P* < 0.001). In the OGD/R + LBP (100 μg/mL) group, TFAM and NRF1 expression levels significantly increased compared to the OGD/R group (*P* < 0.001). However, both TFAM and NRF1 expression levels were significantly reduced in the OGD/R + S100 group compared to the OGD/R + LBP (100 μg/mL) group (*P* < 0.001).

These results aligned with western blot findings, supporting the conclusion that LBP extract confers neuroprotective effects through the SIRT1/PGC-1α signaling pathway.

## 4 Discussion

Stroke is the most prevalent neurological disorder that significantly impacts human health and quality of life. Reperfusion therapy remains the most effective acute-phase stroke treatment; however, it can inadvertently lead to CIRI, causing secondary neuronal damage. Understanding the mechanisms of CIRI and identifying potential therapeutic agents are therefore critical research priorities. Numerous natural compounds have demonstrated anti-ischemic neuroprotective effects in studies globally, with LBP among the promising agents.

This study utilized various concentrations of LBP extract to treat HT22 cells post-OGD/R injury, demonstrating that hypoxia led to reduced cell survival, increased apoptosis, and elevated LDH release. Post-treatment with LBP extract, all three indices showed significant improvement, suggesting that LBP extract has a protective effect on OGD/R-induced neuronal injury. Based on CCK8 assay results, the 100 μg/mL concentration of LBP extract most effectively enhanced HT22 cell survival following OGD/R injury. In other people’s cell experiments, the concentration of LBP extract ranged between 40 and 250 μg/mL, which led us to select this concentration for subsequent experiments [15,16].

ROS is a collective term for reactive oxygen-containing compounds, primarily including oxygen radicals like superoxide anion, hydrogen peroxide, and hydroxyl radicals. Under physiological conditions, intracellular ROS are maintained at low levels, functioning as second messengers in immune defense, tumor suppression, inflammation repair, and signal transduction. However, under pathological conditions, excessive ROS production overwhelms the cell’s clearance capacity, leading to oxidative stress injury from accumulated free radicals, causing cellular and tissue damage [17]. Numerous studies demonstrate that, while blood perfusion is restored in acute cerebral ischemia treatment, reperfusion itself triggers a surge of oxygen free radicals from local brain tissues, inducing oxidative stress and exacerbating injury progression.

Mitochondria are central to cellular energy homeostasis and serve as the primary source of ROS, which are by-products of mitochondrial respiration. Excessively elevated ROS levels can, in turn, damage mitochondria, causing mitochondrial dysfunction and oxidative damage to mitochondrial DNA (mtDNA), ultimately leading to neuronal death [18]. The mitochondrial pathway, or intrinsic apoptosis, occurs when mitochondrial membrane permeability increases in response to stimuli (e.g., free radicals), releasing pro-apoptotic factors (e.g., Bax, Bad) and activating Caspase-3, which leads to apoptosis. Notably, studies have shown that ROS are potent inducers of apoptosis via the mitochondrial pathway and play a critical upstream regulatory role in mitochondrial apoptotic events. In this study, intracellular ROS levels were measured to assess oxidative stress, revealing a significant increase in ROS in HT22 cells after hypoxia-reperfusion. ROS levels were significantly reduced in LBP extract-treated groups, suggesting that LBP extract effectively mitigates ROS generation in HT22 cells subjected to OGD/R-induced injury, consistent with findings from other studies.

SIRT1 is a transcriptional co-activator broadly involved in physiological processes, including cancer, oxidative stress, aging, metabolism, apoptosis, proliferation, and inflammation, largely through regulation of PGC-1α deacetylation [19]. PGC-1α, a transcriptional coactivator, plays a key role in mitochondrial regulation. The nuclear/cytoplasmic localization of PGC-1α is primarily regulated by cellular energy sensors, which, upon positive stimulation by external signals, activate SIRT1 to deacetylate PGC-1α. This deacetylation promotes PGC-1α translocation to the mitochondria, where it binds mtDNA, activates nuclear respiratory factors (NRF1, NRF2), and forms a transcription complex with TFAM, promoting synthesis of mitochondrial enzymes, replication of mtDNA, and mitochondrial maintenance.

Studies show that Dihuang Yinzi attenuates nerve damage by upregulating PGC-α and SIRT1 [20]. In this study, PCR and western blot were used to measure the gene and protein expression of SIRT1 and PGC-1α. Results indicated a decrease in SIRT1 and PGC-1α expression in neurons following hypoxia, which was attenuated by LBP extract treatment. We hypothesize that LBP extract mitigates CIRI by activating the SIRT1/PGC-1α pathway, providing a promising therapeutic strategy for neuroprotection in ischemic injury.

The Keap1-NRF2/ARE signaling pathway, centered on NRF2, is a well-established antioxidant pathway. NRF1 and NRF2 are CNC-bZIP family transcription factors with similar sequences and expression patterns. Like NRF2, NRF1 can bind to ARE elements to regulate the expression of genes involved in endogenous antioxidant responses [21,22]. Under physiological conditions, NRF1 primarily functions to maintain redox homeostasis. Downregulation of NRF1 leads to a significant reduction in ARE-dependent gene expression, resulting in weakened antioxidant capacity [23,24]. TFAM is a key regulator of mitochondrial biogenesis, primarily involved in mtDNA replication and transcription. As downstream targets of PGC-1α, both NRF1 and TFAM co-regulate the respiratory chain complex and support mitochondrial biosynthesis. Reduced expression levels of NRF1 and TFAM indicate mitochondrial dysfunction [25].

In this study, PCR and western blot analyses were used to detect the gene and protein expression of NRF1 and TFAM, and we observed that NRF1 and TFAM expression at lower LBP extract concentrations (25 and 50 μg/mL) was inconsistent. Stable expression levels were observed at 100 μg/mL LBP extract, and treatment with this concentration significantly reversed the decrease in NRF1 and TFAM expression in the OGD/R group. This result was consistent with trends observed in SIRT1/PGC-1α expression. We concluded that LBP extract attenuates CIRI through the activation of the SIRT1/PGC-1α pathway, which subsequently upregulates NRF1 and TFAM.

To validate these results, EX-527 was used to inhibit SIRT1 expression in LBP extract-treated OGD/R cells. With EX-527 interference, cell survival decreased, and apoptosis rates increased compared to the non-interfered group, reducing LBP extract’s cytoprotective effect. Additionally, SIRT1, PGC-1α, NRF1, and TFAM expression levels were reduced, confirming that LBP extract attenuates CIRI via the SIRT1/PGC-1α axis.

In conclusion, our experimental data collectively confirm that LBP extract alleviates hypoxia/reoxygenation injury in HT22 cells, reducing mitochondrial oxidative stress and endogenous apoptosis by activating the SIRT1/PGC-1α pathway. This study provides a foundation for developing LBP extract as a neuroprotective drug candidate, given its high clinical potential due to its origin from the edible *Lycium barbarum* fruit and lack of toxicity.
